# Lectures on the Physical Phenomena of Human Beings

**Published:** 1847-04-01

**Authors:** 


I. Leçons sur les Phénomenes Physiques des Corps Vivants. By Signor *Carlo Matteucci,* Professor in the University of Pisa, &c. Paris, 1847. 12mo. pp. 406, 18 woodcuts.
Lectures on the Physical Phenomena of Human Beings.

II. Electro-Physiological Researches. First Memoir. The Muscular Current. By the same.

III. Electro-Physiological Researches. Second Memoir. On the Proper Current of the Frog. By the same.

IV. Electro-Physiological Researches. Third Memoir. On Induced Contractions. By the same.

[From the Philosophical Transactions for 1845. Part II. Communicated by *Michael Faraday,* Esq. F.R.S.]

In the *Medico-Chirurgical Review* for April 1845, we published a somewhat lengthened article on the interesting and important investigations of Professor Matteucci, contained in his *Traité des Phénomenes Electro-physiologiques des Animaux,* as well as in the *Rapport entre le Sens du Courant Electrique et les Contractions Musculaires dues a se Courant,* published jointly by MM. Longet and Matteucci, and noticed in the *Comptes Rendus* for September 1844.

In that article we announced that the Council of the Royal Society had, on the recommendation of its Committee of Physics, adjudged, to Professor Matteucci, the Copley Medal, on account of the novelty and importance of his researches. To shew his gratitude to the Society for the distinction thus accorded to him, Matteucci communicated, in the three *Memoirs* whose titles stand at the head of this article, some fresh researches on electro-physiological phenomena.

In 1844, the Government of Tuscany appointed Matteucci to deliver, in the University of Pisa, a course of lectures on the physical phenomena of living beings; an interesting subject, for the elucidation of which Matteucci's studies and investigations peculiarly fitted him. These lectures were published in Italy, and have passed through two editions; but they have only recently become known to the English public by the French edition published in the present year, under the direction of the author, who has in it made considerable additions to the matter contained in the second Italian edition.

In these *Leçons,* Matteucci discusses the subjects of the three Memoirs published in the Transactions of the Royal Society, and in the edition now before us he has introduced a notice of all his most recent investigations on Electro-physiology. This of course renders the French edition greatly superior to the editions previously published in Italy.

Professor Matteucci's course consists of twenty lectures, and embraces the following subjects:—

*Molecular Attraction; Capillarity; Imbibition; Endosmose; Absorption*

in animals and vegetables; *Digestion; Respiration; Gaseous Endosmose; Hæmatosis* or sanguification; *Nutrition; Animal Heat; Phosphorescence of organized beings; Muscular Electrical current; Electric Fish; Proper currents of the frog; Physiological action of Gravity, Light, Heat, and the Electrical current; Nervous force; Muscular contraction; Animal Mechanics; Circulation of the blood; Vocal apparatus; Voice; Hearing; Vision.*

In the first lecture the Professor shews that living beings possess the general properties of all the bodies of nature; that the physical forces act on these as well as on other natural bodies; but that organised beings present phenomena termed vital, which, in the present state of our knowledge, cannot be referred to mere physical agencies. During life there is, between the physical and vital forces, a constant struggle, which terminates in the triumph of the former, that is, in death.

Although the study of the influences of physical agents on living beings is a subject of paramount importance in physiology; and although it can be demonstrated that light, heat and electricity exercise, in the interior of living beings, the same kinds of physico-chemical actions which they do in inorganic bodies, yet it must be confessed that, at present, it is quite impossible to explain vital phenomena by reference to physical agency only. No one is more impressed with the truth of this fact than Matteucci.

"With the aid of all this knowledge and of these analogies, dare we hope," says Matteucci, "to obtain a complete explication of all the phenomena of living beings? Alas! such hope for the present would be vain.

"Open an animal, examine the kidneys and liver, and then ask yourselves by what physical force you can explain how the blood, which is carried to an organ, forms bile and urine? Can you, by having recourse to chemical affinities, modified as much as you please, and aided by the peculiar structure of organs and even by the actions of contact,—can you, I will not say comprehend, but even obtain a glimpse of, the means by which the various organs effect the separation and transformation of the constituent parts of the blood, in which all the organic elements are mixed, partly suspended, partly dissolved, and of which they have need to repair their continual losses! What can we say of the functions of the nerves or of generation!"

*Endosmose.*—Passing over *molecular attraction, capillarity,* and *imbibition,* we come to that fertile principle *endosmose,* which forms the subject of Matteucci's third lecture, and to which no less than forty-one pages of the *Leçons* are devoted.

We much wish that our space permitted us to transfer the whole of this interesting lecture to our pages, for it abounds in important matter. It is not a mere *resumé* of what is known on the subject, but contains many novel and ingenious views of paramount interest to the physiologist and practical physician.

In the early part of the third lecture Matteucci falls into an error which is very common with continental, and even with some British, writers; that of ascribing to Dutrochet* the discovery of endosmose.

---

* Science has to deplore the recent loss of this eminent philosopher, who died on the 4th February of the present year, in the 70th year of his age.

The credit of this is, however, really due to our distinguished countryman Mr. Porrett, the present Treasurer of the Chemical Society of London; who, in a letter (dated June 6th, 1816) to Dr. Thomson, the editor of the *Annals of Philosophy*,* describes two "*curious galvanic experiments*," one of which involves the discovery of endosmose. Mr. Porrett is not only the discoverer of the fact just referred to, but he also had the sagacity to suggest its application to the explanation of physiological phenomena. That portion of his paper which relates to the phenomena of endosmose being very short, we feel it our duty, as an act of justice to Mr. Porrett, to reprint it entire, for the benefit of those of our readers, who may not have the time and convenience to refer to the volume which contains it.

"*Exper.* 2.—I took an ounce medicine phial, and with a red-hot rod of iron cut it in a horizontal direction, so as to form the lower part into a small jar. I threw away the upper part and divided the small jar into two equal parts in the direction of its length, so as to make a vertical section of it. The two halves of the jar were then pressed together in their original position, having first interposed a piece of moistened bladder. All the parts of the bladder which protruded beyond the outside of the jar were then cut away; and when this was completed, melted sealing-wax was run down the outer edge of the bladder, and thus the two halves of the glass vessel were firmly united. By this means the inside of the glass jar was divided into two cells, by the bladder interposed between them.

"One of these cells having been filled with water, and left for several hours, was found to have retained the water. The bladder, therefore, was not sufficiently porous to allow the water to filtrate through it. The cell filled with water was now positively electrified, with a battery of 80 pairs of $1\frac{1}{4}$ inch double plates, and a few drops of water were put into the empty cell, so as to cover the bottom of it. This small quantity of water was then negatively electrified. The phenomena which ensued were exceedingly curious and instructive. Independent of the decomposition of a small part of the water, which of course took place in the usual manner, the principal part of it obeyed the impulse of the voltaic current *from the positive to the negative wire*, first overcoming the resistance occasioned by the compact texture of the bladder, so as in about half an hour to have brought the water in both cells to the same level, and afterwards overcoming the additional resistance occasioned by the gravitation of the water, by continuing to convey that fluid in to the negative cell, until its surface in that cell was upwards of $\frac{3}{4}$ of an inch higher than in the positive cell. A much greater difference of level might doubtless be obtained by operating with a larger apparatus, and for a longer time; but the results are perfectly conclusive when the experiment is performed on the small scale in which I tried it.

"I have repeated the above experiment several times, and invariably found the liquid, whatever it was, descend on the side positively electrified, and ascend on that negatively electrified, chemical changes at the same time going on, as in the celebrated transfer experiments of Sir H. Davy; but those experiments could not show the mechanical action of the voltaic current, consequently only the chemical action was observed in them. To render the mechanical action evident, it is an indispensable condition that there should be interposed between the positively and negatively electrified liquids a body which, although porous, is yet sufficiently compact to prevent filtration taking place in common circumstances. Bladder answers this condition. I do not think, however, that it does so as well as filtering-paper that has been prepared in the following manner, suggested to me by my very ingenious friend Mr. Wilson, of Guy's Hospital:—Spread the white of an egg thinly upon filtering-paper; then immerse the paper into boiling

---

* See the *Annals of Philosophy* for July, 1816, Vol. 8, p. 74.

water, so as to coagulate the albumen; it is then well adapted for these experiments. Thick paper of a very compact texture would probably do without this preparation; but I cannot state this positively, not having tried it.

"I think that by the above experiment I have demonstrated the existence of a power not before noticed in the voltaic current, namely, that of conveying fluids through minute pores not otherwise pervious to them, and overcoming the force of gravity.

"Is not this electro-filtration, jointly with electro-chemical action, in constant operation in the minute vessels and pores of the animal system?

"I wish that some person well versed in the sciences of anatomy, chemistry, and electricity, would answer this question. I am not qualified to attempt its solution, being a stranger to the first mentioned science, and possessing but a moderate knowledge of the other two; and it appears to me that only a proficient in all should venture to propose any new physiological opinions, but I cannot help thinking that an affirmative answer to the above question is capable of a good defence.

"To those who may be inclined to repeat the preceding experiment, it may be useful to mention that, by letting fall from a dropping tube a little sulphuric acid into the cells of the battery occasionally, its action is prolonged, without the trouble of renewing the liquid in the cells, or the inconvenience of disturbing the whole arrangement, the partial action of this dense acid on the plates is prevented by stirring the liquid afterwards with a little stick." P. 76.

After this *réclamation* we proceed with our notice of the *Leçons*. The simple fundamental fact relative to endosmose is thus stated by Matteucci.

"Here is a glass tube, whose lower extremity is expanded into the form of a funnel, and is closed by a piece of bladder. This instrument is called an *endosmometer*. If we pour into it an aqueous solution of either gum or sugar and then plunge the closed extremity into water, we shall observe that, notwithstanding the excess of pressure of the column of liquid, the water continually passes into the tube by filtration through the membrane. The liquid within the tube thus becomes elevated to a certain extent and may even flow over at the upper extremity: at the same time, however, a certain quantity of mucilaginous or saccharine liquid escapes from the tube through the bladder and mixes with the water; but the quantity is necessarily less than that of the water which passed through the membrane from without inwards. Dutrochet has applied the term *endosmose* to the first of these phenomena (that is to the passage of the fluid from without inwards), and that of *exosmose* to the passage of the fluid from within outwards.

"Membranes produce endosmose until they begin to putrefy, when the phenomenon ceases, and the liquid, which had been elevated in the tube, falls and filters through the membrane."

The phenomena of endosmose are not confined to membranes, or even to organized substances. Plates of slate or of baked clay produce, though in a weaker degree, the same phenomena.

The nature of the liquid has a considerable influence on the process.

"It is a curious fact," says Matteucci, "that the slightest trace of sulphuretted hydrogen modifies the production of this phenomenon, even with the most active liquids: whereas other acids, as the hydrochloric and nitric, have no effect on it.

"When compared with water, all animal liquids produce endosmose with much energy, except those contained in the large intestine. These probably become exceptions on account of the sulphuretted hydrogen which they contain."

Matteucci then briefly notices Dutrochet's experiments on the velocity of endosmose with different fluids, and mentions the curious fact, that certain acids, when mixed with water, change the direction of the current. After some remarks on the force of the endosmotic current and the insufficiency of theory to account for the phenomenon, Matteucci observes—

"What we have now stated is sufficient to convince you that this phenomenon is perhaps the most important of physical facts, with respect to its applications to the functions of living bodies.

"Microscropic observation has now put beyond a doubt that, in all tissues, vegetable or animal, and in those liquids which are produced by the alteration of organised and living beings, there is constantly found, at a certain epoch of their formation, microscopic corpuscles, which have a peculiar and characteristic form, and are called *elementary, or primitive cells.* These bodies consist of an excessively fine membrane, have a spherical form, and enclose a liquid. On the inner side of the membrane is a small organised body, called the *nucleus* or *cytoblast.* The cells float at first in a liquid which Schwann has named *cytoblastema,* and ultimately become included in, and almost confounded with it, when this liquid becomes more or less dense. In different tissues, the elementary cells are more or less closely approximated to each other; the *cytoblastema,* or intercellular substance, is invariably the bond of union between the cells. Hereafter we may perhaps return to this important subject, which we have now only glanced at, in order to render more evident the importance of the phenomenon of endosmose. The life of the elementary cells certainly forms the part which is the most essential to the development and preservation of the tissues of living bodies; and, since these cells are found under conditions favorable to endosmose, we can assign no reason why it should not take place. A vessel filled with a liquid, and placed in the midst of another liquid, may act on the outer one, receive the surrounding liquor, and reject the one it had previously contained, by operating in a manner analogous to endosmose.

"We must, however, confess that hitherto very few investigations have been undertaken with the view of making such applications of the phenomenon of endosmose to physiology as it appears to be susceptible of. To do this it was necessary to vary the liquids between which endosmose takes place, and to select the membranes, so that we might always keep as close as possible to the conditions under which the analogies between the phenomenon and those which take place in the interior of living bodies have been observed. This I undertook to effect, in conjunction with Professor Cima; and I shall now bring before you the result of our researches. The membranes which we submitted to experiment may be divided into three classes: the first includes the skin of the frog, the torpedo, and the eel; the second, the stomach of the lamb, the cat, and the dog, and the gizzard of the fowl; and the third, the bladder of the ox and of the pig."

Matteucci then proceeds to explain the apparatus employed, and the conditions under which the experiments were made, to determine the influence which the membranes of these three classes exercise on endosmose.

One of the most remarkable facts discovered by MM. Matteucci and Cima is the marked influence exercised on the phenomenon of endosmose by the position of the membrane interposed between the two liquids. Thus, in the case of the fresh mucous membrane of the bladder of the ox, deprived of the muscular coat, this influence was very manifest,

"when this membrane was employed, and a solution of sugar introduced into the interior of the two endosmometers, the height at which the liquids arrived in the tubes was, when the internal surface of the membrane was in contact with the saccharine liquid, 80 and even 113 millimetres in the usual space of two hours; but it was only 63 or 72 millimetres when the position of the membrane was reversed. The current of endosmose then is promoted in this instance from the external to the internal surface of the membrane. The contrary effect is obtained with the solution of gum arabic. The elevation is 18 and sometimes only 7 millimetres, when the internal surface is turned towards the interior of the instrument, when it contains the gum solution; whereas, when the membrane is arranged the reverse way, the elevation is 52 millimetres, or, in some cases, 20 millimetres."

The general conclusions drawn from the various experiments made by MM. Matteucci and Cima on this subject are thus stated by Matteucci:—

"1st. The membrane interposed between the two liquids, in the phenomenon of endosmose, has a very active share in the intensity of the endosmometric currents, as well as in its direction.

"2nd. There is in general, for each membrane, a certain position in which endosmose is the most intense; and the cases are very rare in which, with fresh membrane, endosmose takes place equally, whatever be the position of the membrane to the two liquids.

"3rd. The direction which is most favourable to endosmose through skins is, usually, from the internal surface to the external, with the exception of the skin of the frog, in which, endosmose between water and alcohol is promoted from the external to the internal surface.

"4thly. The direction favourable to endosmose through stomachs and bladders varies much more than with skins, according to the different liquids.

"5thly. The phenomenon of endosmose is closely allied to the physiological condition of the membranes.

"6thly. With membranes dried or altered by putrefaction, either we do not observe the usual difference belonging to the position of their surfaces, or endosmose no longer takes place."

The following extract forms the conclusion of the third lecture of Matteucci's very interesting work.

"It is by endosmose that physiologists now explain the nutrition of the ovules in the oviducts of mammalia, and how the sacs, which contain the sperm of the cephalopodous molluscs open immediately they are brought into contact with water.

"A cell is the elementary organ of all animal and vegetable tissues, and cell-life involves an act of endosmose: this shows how much the phenomenon of endosmose is still in want of being more completely studied, in order that we may be enabled to make of it all the applications of which it is susceptible. I cannot conclude this lecture without referring to the recent experiments of Poiseuille, made with the view of explaining by endosmose the purgative action of certain substances. He found that there was endosmose through animal tissues from the serum to Sedlitz water, and to the solutions of sulphate of soda, and common salt. Now this is precisely what happens when we use these medicines internally. The rejected excrements contain an abundant and unusual quantity of albumen: in this case we must admit that endosmose takes place through the capillary vessels of the intestine, from the serum of the blood to the saline solution introduced into the alimentary canal.

"But, to remove all doubt of the propriety of Poiseuille's applications of this fact, it was necessary to demonstrate that endosmose takes place when one of the liquids is in motion, and is continually renewed.

"This has been recently done by Dr. Bacchetti, who has shewn that the rapidity of endosmose is considerably augmented when one of the liquids was continually renewed. This result, moreover, is in accordance with the principles of the theory of endosmose: the exchange of liquids constantly effected through the membrane, leads to the suspension of the action of endosmose; or, in other words, the conditions for the production of the phenomenon are so much the better preserved, as the liquids remain longer without mixing. Poiseuille has also shewn that endosmose ceases to take place in a membrane after a certain time of action, but that we may restore to the membrane this property by submitting it to the action of other liquids. The most remarkable fact discovered by Poiseuille is that of the influence exercised by hydrochlorate of morphia. This body added to saline solutions weakens very considerably the endosmose from the serum to the solution; and ultimately changes the direction of the current. This fact has been confirmed by Dr. Bacchetti. How can we make an entire abstraction of this fact in the explanation of the action of morphia and the preparations of opium in diarrhœa, and of the constipation which they produce?"

*The Muscular Current.*—In the paper on the muscular current, presented by Matteucci to the Royal Society and printed in their Transactions, the results obtained from his different experiments are thus summed up.

"In the first place, the intensity and duration of the muscular current are independent of the nature of the gas which envelops the muscular pile. Secondly, this current, as I have already shown from the commencement of my researches, is altogether independent of the cerebro-spinal nervous system, and the circumstances which exercise a marked influence upon its intensity are respiration and the sanguineous circulation. Thirdly, those poisons which seem to act directly upon the nervous system, have no influence upon the muscular current; among these I would mention hydrocyanic acid, morphine and strychnine. Fourthly, sulphuretted hydrogen has a marked influence in diminishing the intensity of the muscular current. Fifthly, the intensity of the muscular current varies according to the temperature in which the frogs have lived a certain time; it is needless to observe that this result is not discoverable except in those animals which, like the frog, necessarily take their temperature from that of the medium in which they live. Sixthly, the intensity of the muscular current increases in proportion to the rank the animals occupy in the scale of beings, while the duration of this current, after the death of the animal, is in an exactly inverse ratio.

"Comparing these conclusions with those generally admitted by physiologists, and drawn from a great number of experiments on the vital properties of muscles, it is impossible not to perceive that the property of the muscles, immediately connected with the muscular current, is that which Haller calls irritability, and which at the present day, I believe, physiologists designate by the name of organic contractility, or simply contractility."

Our readers will not fail to perceive that the conclusions drawn by Matteucci from his experiments are opposed to the views of Dr. Marshall Hall. The Italian Professor regards the irritability of muscular fibre as inherent; whereas Dr. Hall considers it as derived from what he calls the true spinal system.

"With regard to the manner of representing the origin of the muscular current," says Matteucci, "I find, in my present experiments, a confirmation of the opinion I set forth in my preceding ones. The chemical action which goes on in the nutrition of the muscle, principally that which takes place in the contact of the arterial blood with the muscular fibre, is in all probability the source of this electricity in the muscles."

Some of the recent investigations of Liebig have reference to the origin of the muscular current. The celebrated Giessen Professor has at last succeeded in demonstrating the existence of free lactic and phosphoric acids in the muscles of animals. He thinks that his results "explain the quick re-action of the muscles," and he adds that, now that "we know that there exists, in so large a portion of the body of animals, an acid liquor, which is only separated from an alkaline fluid (the blood and the lymph) by very thin membranes, we may, I think, explain several electrical phenomena observed by Matteucci and other physiologists upon the bodies of dead (and living) animals." (See *Chemical Gazette*, Feb. 1, and Feb. 15, 1847; also *Comptes Rendus*, Jan. 18th, 1847.)

*The Proper Current of the Frog.*—In our review of Matteucci's *Traité des Phénomenes Electro-physiologiques* (*Medico-Chirurgical Review* for April 1845), we protested against Matteucci's assumption that the frog had any peculiar electric current; and we did so because it appeared to us that the assumption was in opposition to everything then known respecting the organization and physiological relations of animals; and we observed that we could not "for a moment admit the probability of the frog possessing a peculiar electric current, unendowed as this animal is with any peculiar organs or electric apparatus." And we further stated our confident belief that, "whatever currents may be detected in the frog, the same will be found to exist, in some degree of intensity, in other animals."

The accuracy of our view is now fully established by the more recent investigations of Matteucci himself, detailed in his second Memoir, subsequently published in the *Philosophical Transactions.*

From the additional experiments, referred to in the Memoir just quoted, made with the view of clearing up certain points which had been left in an unsettled state, in the *Traité*, Matteucci concludes,

"That the proper and the muscular current are in general subjected to the same laws, and that both these currents vary in the same sense, under the same circumstances."

Feeling the importance of solving the question why the proper current should belong exclusively to the frog, Matteucci next directed his attention to this point, and arrived at the following

"Generalization of the fact of the proper current of the frog; the current is directed within the muscle from the tendon to the superficies.

"It remained for me to extend this fact to its operation upon the muscles of warm-blooded animals, and the experiments accorded in such a manner as to leave no possible doubt."

His experiments were made on fowls, pigeons, rabbits, and dogs; and their results were such as to establish the correctness of the position we took up in the review before quoted; and we therefore claim for ourselves the credit, small though it be, of having denied the speciality of the proper current in the frog, and of having asserted our belief of its universality in animals. In his *Leçons*, Matteucci thus clearly states the conclusions at which he has recently arrived.

"Recently, by studying more attentively the proper current, I have satisfied myself that it is a phenomenon which appertains to all animals. Here is the enunciation of the fact: in every muscle endowed with life in which the tendinous extremities are not equally disposed, there exists a current directed from the tendon to the muscle, in the interior of the muscle. All animals have muscles in which one tendinous extremity is narrower than the other; which at one part forms a kind of cord, and at the other becomes broader and ribbon-like. In the frog and many other animals, the gastrocnemius has this character: in birds, the pectoral muscle presents this arrangement. When we form a pile with these muscles, we find that a current circulates in the muscle, from the tendinous extremity to the muscular surface.

"In arranging this pile, we must carefully avoid exposing the internal part of the muscle, and we must especially place one element in contact with another, in such a manner that the tendinous extremity touches the surface of the muscle, and never the interior: indeed, the latter ought to be as far as possible from the tendon. Without this precaution, there will be, in the circuit, the muscular current, which, being directed from the interior to the surface, would have a direction precisely the reverse of that of the proper current. Having thus ascertained the conditions on which the proper current depends, I think that I may generalise its origin and connect it with the muscular current. This community of origin is principally demonstrated by the identity of action which the different circumstances that modify the organism and the life of animals exercise upon the muscular current. In fact, whether the current be muscular or proper, the action exercised on it by heat, narcotics, sulphuretted hydrogen, and the degree of integrity of the nervous system is the same.

"Anatomists have lately demonstrated that the elementary muscular fibres are immediately continuous with the tendinous fibres, and that the sarcolemma which invests the muscle, ceases abruptly where the tendon begins. We may, therefore, with some probability, consider the tendon as being in the same electric condition as the interior of the muscle; and, therefore, when we form, by means of a good conductor, a circuit or communication between the tendon and the sarcolemma, we put into circulation a portion of the muscular current."

*On Induced Contractions.*—In our review, before referred to, of Matteucci's *Traité*, we noticed the *physiological phenomenon produced by a muscle in contracting*, and proposed to call it electric sympathy, Matteucci now terms it *induced contraction*, or *muscular induction*. The subject, though important to the physiologist, is very obscure. In his later memoir on it, inserted in the *Philosophical Transactions*, Matteucci first examines the question whether electricity is evolved during the contraction of a muscle; and concludes that it is not. After examining various physical theories of induced contraction, he concludes by admitting that "we cannot give a satisfactory explanation of the phenomenon of induced contraction by recurring to electricity or any other known causes," and infers that "induced contraction is only a new phenomenon of nervous force."

We must now conclude our account of Matteucci's Lectures and Memoirs. In noticing the former we have been obliged to limit our extracts and observations to a few only of the subjects treated of. But, as we have enumerated the various topics which the author successively considers, our readers will be able to form a very fair idea of the extent and scope of this most interesting and instructive work; and we trust that the extracts which we have made from it will justify our opinion of its highly meritorious character. It is alike valuable and interesting to the general reader and the professional man,—to the natural historian and natural philosopher,—to the physiologist and the practical physician.

With the exception of Magendie's Lectures (published in Paris in 1842 in 4 volumes 8vo.), we are unacquainted with any other modern work, besides that of Matteucci, which is expressly devoted to the physical phenomena of living beings, yet the subject is of growing importance, and loudly calls for further investigation. One reason why its literature is so scanty is, perhaps, the paucity of persons who combine, in one individual, a sufficient acquaintance with both physical and physiological sciences, to qualify him for the investigation: physics and physiology being usually regarded as entirely different branches of study. In the case of Magendie we have an instance of a physiologist successfully pursuing physical researches with the view of applying physical agencies to the explanation of physiological phenomena. Matteucci, on the other hand, is a natural philosopher, a professor of the physical sciences, who has extended his investigations to organized bodies, in order to ascertain whether many of the phenomena presented by living beings, and called vital, may not be, in reality, due to physical influences. The subject, therefore, has been regarded by those two eminent philosophers from opposite points of view; and the work of the one is consequently not a substitute for that of the other.

Some of our readers may perhaps be glad to learn that an English translation of this work, made under the superintendence of, and annotated by, Dr. Pereira, has been announced, by Messrs. Longman and Co., for immediate publication.

